# Supplementary material for: Are carbon emissions trading and green financial instruments synergistic? -Comprehensive quantitative research based on content analysis
Source: PLoS One. 2024 Mar 7;19(3):e0298601. doi: 10.1371/journal.pone.0298601 (PMC10919601; doi:10.1371/journal.pone.0298601)
Supplement: S1 Data — (ZIP) [file pone.0298601.s001.zip › Data/地级市Z_F工作报告.zip!/╕ú╜¿/─■╡┬╩╨/└┤╫╘╣1⁄2╓┌║┼í╛┬φ┐╦╩2╛▌═°í┐_2003.docx]

各位代表：

现在，我代表市人民政府向大会作政府工作报告，请予审议，并请市政协各位委员和其他列席会议人员提出意见。由于《政府工作报告》全文已印发给各位代表和委员，这里，我仅就报告的主要内容作简要汇报。

一、2002年政府工作回顾

2002年，我市各级政府认真贯彻十六大精神和“三个代表”重要思想，在省委、省政府的领导下，按照市委的部署，紧紧围绕构建三条战略通道，扎实推进第二层面发展。经过全市上下共同努力，较好完成市一届人大三次会议确定的各项任务。全年国内生产总值244.62亿元，比上年增长9%。三次产业结构由上年的29.8：32.6：37.6调整为28.0:33.4:38.6。社会消费品零售总额83.7亿元，增长8.7%。地方级财政收入10.12亿元，增长11.5%。年末金融机构各项存款余额137.35亿元，增长26.0%；各项贷款余额110.30亿元，增长21.0%。一是农业结构调整步伐加快，农业农村经济继续发展。二是工业发展速度继续加快，企业经济效益有所提高。三是重点项目建设成效显著，固定资产投资继续增长。四是体制改革扎实推进，外经贸工作取得可喜成绩。五是城市建设管理力度加大，中心城市新形象进一步凸现。六是城乡居民生活水平明显提高，社会保障工作得到加强。七是科教兴市取得积极成果，社会事业稳步发展。八是精神文明建设卓有成效，民主法制建设继续加强。

过去一年的发展成就，是在世界经济增长明显减缓，国际国内市场竞争更加激烈，我市遭受第16号台风袭击、受灾较为严重等情况下取得的。这些成绩凝聚着全市人民的智慧和力量，体现了各个方面的帮助和支持。在这里，我代表市人民政府，向全市人民，向给予我们支持与监督的人大代表、政协委员、各民主党派和工商联、各人民团体、社会各界人士，向大力支持我们工作的驻宁部队、武警、民兵预备役官兵、公安政法干警、离退休干部、港澳台同胞、海外侨胞表示衷心的感谢和崇高的敬意！

在充分肯定成绩的同时，我们也清醒地认识到，在经济社会发展中，还存在一些困难和问题。主要是：农产品价格持续低迷，农民增收难问题比较突出；工业化和城镇化水平有待提高，经济增长缺乏支柱产业的支撑；项目前期工作深度不够，固定资产投资有弱化趋势；城乡差距、地区差距继续扩大，县乡经济基础还较薄弱；就业和再就业形势较为严峻，城乡部分群众生活还比较困难；政府职能、管理方式、思想观念、工作作风要继续转变，投资软环境仍不理想；新生财点培植成效不大，财政比较困难；高层次实用人才缺乏，社会事业基础薄弱；社会治安状况仍不尽人意，一些消极腐败现象仍然存在。对此，我们将高度重视，在今后的工作中，采取有力措施，努力加以解决。

二、扎实做好2003年工作

2003年是学习贯彻党的十六大精神、推进全面建设小康社会的第一年。做好今年的政府工作，对于实现我市“十五”计划和全面建设小康社会目标，具有十分重要的意义。

根据市委的部署，今年工作的总体要求是：以邓小平理论和“三个代表”重要思想为指导，认真贯彻党的十六大以及全国、全省“两会”精神，按照市委一届六次全会的部署，始终以全面建设小康社会统揽工作全局，始终把加快发展作为富民强市的第一要务，着力构建三条战略通道，实施三大战略，加快四大基地建设，推动宁德向第二层面发展。继续深化改革，扩大开放，加大固定资产投资力度，改善发展环境，推进经济结构调整，加快产业升级，积极发展农业和农村经济，致力提高工业化、城镇化水平，壮大经济总量，促进国民经济持续快速健康发展和社会进步，实现速度、结构、质量、效益相统一。正确处理改革发展稳定的关系，切实做好就业和再就业工作，完善社会保障体系，提高城乡居民生活水平。大力实施科教兴市和可持续发展战略，加快社会主义民主法制建设，推进社会主义物质文明、政治文明和精神文明建设。

按照这一总体要求，今年我市国民经济和社会发展的主要预期目标是：国内生产总值增长9%，力争更快更好些；全社会固定资产投资与上年持平；社会商品零售总额增长9%；地方级财政收入同口径增长10%；外贸出口增长12%，实际利用外商直接投资增长9%；居民消费价格涨幅与上年基本持平；城镇居民人均可支配收入增长5—6%；农民人均纯收入增长4—5%；城镇登记失业率控制在4.5%以内，人口自然增长率控制在7.5‰以内。为此，今年必须着重做好以下10个方面的工作：

（一）加强农业和农村经济工作，促进农业增效农民增收

优化农业和农村经济结构，推进农业产业化。一是培育优势主导产业。着力建设特色农业“两带一区”，促进优势农产品和特色农产品向优势产区集中。保护和提高粮食综合生产能力，努力扩大优质稻种植面积。加快水产、茶叶、食用菌、水果、药材等传统农业改造步伐，构大畜牧产业，培育园艺等新经济增长点，大力发展抗风浪大型深水网箱、围网养殖和淡水养殖。二是着力发展农产品加工业。鼓励和引导乡镇企业、民营企业和外商投资兴办农产品加工业。培育扶持全市90家规模以上农产品加工企业。三是发展壮大龙头企业。加大对现有22家省、市级农业产业化龙头企业扶持力度，支持这些企业扩大生产规模。四是强化农业招商引资工作。加大农业招商引资力度，加强宁台农业合作与交流，力争在农产品加工项目的招商引资方面有新突破。

加快科技兴农步伐，努力提高农民非农收入。全面组织实施农业科技“三个十工程”。加快建立农产品质量标准和检测检验体系，引导农民按照质量标准组织生产，力争今年全市有更多的产品获省名牌农产品、绿色食品标志。重视畜牧业疫病防治工作，建立农产品安全体系。清理对农民进城务工就业的不合理限制，逐步降低农民进城门槛，有重点地引导农民进入小城镇。加强对进城农民的服务和管理，抓好农民职业技能培训和信息中介服务工作。加快乡镇企业的调整和改革，拓展农村就业领域，促进农村劳动力转移，增加农民非农收入。

加强农业基础设施建设，巩固提高脱贫致富成果。抓好农业基础设施的维护和建设。抓紧宁德市城区防洪挡潮工程和上白石水利枢纽项目前期工作，力争动工建设。大力推广扶贫小额信贷，加大扶贫开发力度，突出抓好45个省定和28个市定重点村的挂钩扶贫工作，切实解决3.13万绝对贫困人口和3.2万低收入人口的温饱问题。努力完成5000人的造福工程、山体滑坡险情村搬迁任务，继续改善“造福工程”搬迁户和已上岸定居的连家船民以及库区移民生产生活条件。

推进农村改革和政策落实，健全完善农村社会化服务体系。在完善家庭承包经营责任制的基础上，按照依法、自愿、有偿的原则，推进农村土地承包经营权流转。全面推行农村税费改革，完善涉农价格收费公示制度。大力加强农村社会服务联动网建设，健全完善社会服务体系和市场信息体系。

（二）构筑工业经济发展平台，全面推进“工业富市”

在培育壮大产业规模上取得突破。一是提升一批。扶持电机电器、食品加工、建筑建材、船舶修造、医药化工、三车配件等六大产业做大做精做强，开发上下游产品，延伸产业链，壮大产业群。二是引进一批。争取引进省内外有实力的大型企业到我市投资办厂，鼓励我市中小企业参与大企业的专业化分工。三是改造一批。抓好柘荣广生堂GMP异地搬迁等10大技改项目实施。

在抓龙头创名牌上取得突破。争取组建电机电器、建筑建材、医药化工等企业集团，力争培育亿元以上企业15家。大力推进ISO9000系列质量体系认证工作，引导企业按国际标准组织生产管理，争创一批名牌产品。

在构筑工业载体上取得突破。坚持总体规划、分步实施、滚动发展的原则，降低电价、地价，调整充实扶持工业园区发展的优惠政策。采取开放式经营、封闭式管理模式，办好闽东、福安、福鼎、霞浦四个工业园区，加快山区五县工业园区发展步伐，做到开发一片、建成一片、投产一片、收益一片，特别要力争闽东工业园区第一期工程开工建设。

在引进工业大项目上取得突破。坚持大中小项目一起要，内外资一起引，劳动、资金、技术密集型产业同发展的原则，大力实施项目带动战略，集中力量抓好一批工业生产性项目，特别要力争冶金、石化下游产品、火电厂等一批大型临海工业项目在我市布点建设，以大项目带动形成新的主导产业。

在海洋产业开发上取得突破。全面实施海洋功能区划，抓紧完成《宁德市港口总体布局规划》编制工作，妥善处理保护与开发的关系，促进海域合理开发利用和海洋环境保护。大力发展海洋渔业、临海工业、港口运输、海洋高新技术等产业，重点抓好大型工业项目专用码头、漳湾、三沙、沙埕等港口资源的开发利用。

（三）继续培育和扩大内需，拉动经济增长

继续加快重点工程建设。初步考虑安排93个项目，其中重点建设项目33个、预备重点建设项目22个、重点前期项目20个、预备前期项目18个。一是继续实施“大交通、大港口、大腹地”战略。把福宁高速公路、温福铁路、“一个半小时交通网”三个项目作为重中之重的项目。确保福宁高速公路主线全线通车。开工建设温福铁路及“一个半小时交通网”5条公路，加快京福高速公路古田连接线项目前期工作，完成城澳万吨码头建设任务。抓好福宁高速公路至港口连接线公路规划工作，重点加快疏港公路建设。二是加强城市基础设施建设。续建和建设中心城市、福安、福鼎、霞浦新城区路网项目。开工建设宁德污水处理厂、垃圾填埋场等一批城市供水排水、园林绿化、污水垃圾处理项目。三是加快能源项目建设。抓好穆阳溪梯级电站二级周宁电站、城乡电网改造等一批能源项目的续建和扫尾，开工建设洪口、牛头山、双口渡水电站等一批能源项目。全面完成区域流域规划工作，出台《宁德市水电建设管理办法》，做好新一轮电气化县建设工作。四是加快社会公益事业项目建设。重点建设和续建宁德学院、体育中心、广电中心、文化中心、宁德医院、闽东医院、妇女儿童活动中心。五是积极做好项目开发储备工作。建立项目推介新闻发布会制度，实行重大项目前期工作领导挂钩责任制。六是多渠道筹措建设资金。积极争取国债和国家、省项目建设资金及金融部门资金。加快实行以项目带资金的管理体制，鼓励和引导民间资金投入国家鼓励的基础设施项目建设。七是加强重点项目组织协调工作。强化进度、质量和投资三大控制，搞好项目稽察和招投标市场整顿工作，确保工程质量。

进一步扩大消费需求。一是千方百计增加城乡居民收入。二是改善消费环境。三是积极培育消费增长点。四是开拓农村市场。

（四）加快中心城市建设，推进县域经济发展

大力推进城镇化。一是加强城市规划。着手调整修编宁德市城市总体规划，加大执法力度，维护规划的严肃性。二是加快滨海城市建设步伐。加快中心城市新区开发，加快东侨至赛岐滨海大道项目前期工作。加快福安、福鼎、霞浦新城区规划和基础设施建设步伐，积极稳妥地推进旧城成片改造。三是加快县城和中心城镇建设。扩大山区县城规模。做好即将动工的温福铁路新站点和高速公路沿线互通口13个乡镇及沿国道、省道15个乡镇的科学规划。合理调整中心村布局。四是做好经营城市文章。继续抓好国有经营性土地使用权等城市资源公开招标拍卖和挂牌交易，加快市政公用设施市场化运作、企业化经营步伐。五是加大产业和人口聚集力度。六是强化城市管理。继续开展创建文明城市活动，切实加大城区“五乱”专项治理力度，强化市民素质教育，提高“新、绿、亮、洁”水平。

积极推动县域经济发展。加快市级简政放权步伐，尽最大可能扩大县级经济管理自主权。市直部门要切实转变工作方式，把工作的着力点放在培育市本级新经济增长点，千方百计壮大市本级经济实力。沿海县（市、区）要做强做大主导产业，加快形成优势区域和产业带，增强沿海对山区的辐射和带动作用。山区县要大力发展资源转换型、传统产业升级型、农副产品加工型、生态型产业，增强山区自我发展能力。同时，加强对县乡公路、电力等基础设施建设的支持与投入，进一步落实扶持山区、老区和民族地区加快发展的各项政策。

加快开发区发展步伐。重新修编开发区总体规划，进一步明确东侨、赛岐、三都澳、福安畲族四个开发区定位和功能，发挥优势，促进开发区产业科学合理布局。积极创新开发区经营模式，理顺开发区管理体制，实现开发区经营主体多元化。完善开发区基础设施建设，降低进入门槛，做好招商引资和项目落地等工作，使之尽快形成规模和示范效应。

（五）加强制度创新，完善市场经济运行机制

继续加快国有企业改革步伐。抓紧研究设立国有资产管理机构，推进经营性国有资产的授权经营。对改革不到位的国有企业，重新制订改革方案，按照建立现代企业制度要求，加大产权制度改革力度，完善法人治理结构。加快内贸、外贸等非工业系统企业改革步伐。同时，扎实推进医药卫生、粮食购销和电力体制改革。

大力扶持非公有制经济发展。进一步完善和落实鼓励民营经济发展政策措施，着力解决民营企业在融资、用地、出入境等方面存在的困难和问题。有重点地引导民营企业投资教育、卫生、体育、旅游、特色农业、环保等产业，扶持一批民营企业兼并、收购、控股、参股国有企业。

做好资本营运文章。加快剥离、激活经营困难的国有企业优质资产和行政事业单位资产。健全完善土地收购储备中心，垄断土地一级市场，放开搞活土地二级市场。推进已建成的项目和在建项目变现增值，引导电机电器、交通运输等优势产业和企业组建股份制公司，并力争上市融资。

整顿和规范市场经济秩序。抓住直接关系人民群众安全、健康等领域中存在的问题，继续加强专项整治，严厉打击制假售假行为。推进餐桌污染治理，做好食品安全卫生工作。强化安全生产管理，遏制重特大事故发生。加快社会信用体系建设，创造良好的市场环境。

（六）拓展对外经贸，进一步提高对外开放水平

加快对内联接步伐。积极主动接受沿海地区尤其是浙江经济的辐射，主动承接区外的资金、设备、技术、人才等要素的转移，全力争取温州等发达地区的企业到我市设点办厂。充分发挥我市毗邻浙南、福州的优势，开展旅游推介促销，做好边界贸易文章。

加大招商引资力度。认真办好“6·28”第四届闽东招商节，积极组织参加厦门“9·8”投洽会等重大活动。在加强工业、农业、商贸、交通基础设施项目吸引外商投资工作的同时，推进旅游、教育、医疗、中介服务、公用事业等领域的引进外资工作。改进招商方式，设立招商促进中心，组织到温州、昆山等地开展小型招商和特色招商活动，抓好产业招商。切实办好现有外商投资企业，促进企业增资扩股。落实招商引资奖励机制，加强已签约项目的跟踪，促进项目升级和到资。充分发挥外事、侨务部门和驻外机构的“窗口”、桥梁作用，进一步搞好外引内联工作。

大力推动出口增长。加快传统出口商品升级换代，培育一批科技含量高、市场竞争力强的出口主导产品。认真推动外贸主体多元化，鼓励外经贸企业开展环境、产品质量和安全认证，积极应对国际非关税壁垒。大力推进电机电器、茶叶、食用菌、水产品等出口基地建设，壮大出口企业。巩固现有国际市场，开辟新兴市场，拓宽出口渠道。同时，积极稳妥地实施“走出去”战略，支持有比较优势的各类所有制企业特别是民营企业到境外投资办厂。

加强宁德与台港澳侨经贸合作。加大对台招商引资力度，特别要抓住台湾新一轮产业转移的新机遇，争取更多台资企业到宁德落户。充分发挥三沙、沙埕等港口的对台贸易窗口作用，积极争取恢复对台劳务输出，拓展境外劳务市场。推进宁德与港澳经贸合作，加强同海外华侨华人及社团的联系和沟通。

切实加强投资软环境建设。认真贯彻落实市委、市政府《关于进一步优化投资软环境的若干意见》，下决心解决外商反映强烈的历史遗留问题，开展外商投资软环境专项治理和监测评价工作。加强投资服务中心建设，继续减少和规范审批行为，全面推行承诺制和代理制。严格规范各类执法检查、各种税外收费，整治“三乱”，加大对破坏软环境行为的查处力度。

（七）以现代流通业为突破口，加快服务业发展

加大旅游产业开发力度。制定旅游发展总体规划，引导和支持各类经济组织参与开发旅游资源，管理经营旅游景点、景区及宾馆等旅游服务设施。开展宁德旅游宣传促销年活动。以太姥山国家级风景名胜区为龙头，着力加快滨海旅游线、绿色山水生态旅游线、民俗宗教文化和畲族风情旅游线路建设，抓好10个旅游精品工程。

建立健全营销体系。大力支持和推动连锁经营、物流配送、多式联运等现代流通业发展。培育发展电子商务等无形市场，积极引导城市超市、专卖店、购物中心向中心城镇延伸。积极引进国内外知名零售企业进入宁德设立连锁分店、采购和物流配送机构，力促宁德产品纳入其商品采购目录，进入国内外市场分销渠道。

拓宽服务业发展空间。积极应用现代经营方式和信息技术改组改造商贸、交通运输等传统服务业。加快社区服务、物业管理、会展业、信息咨询、中介服务、教育培训、文化娱乐、卫生保健、体育健身等新兴服务业发展。

加快市场体系建设步伐。加快“三场三街”在建项目建设进度，推动农贸市场超市化改造。同时，大力拓宽市外市场，培育和拓展“宁货”在全国、全省重点地区的营销网络。

积极引导和规范房地产业健康发展。加强房地产开发的规划指导，促进房地产业健康发展。进一步深化住房制度改革，全面放开住房二级市场。着力激活经济适用房市场，强化公积金管理，继续解决机关干部住房难问题。

（八）认真做好财税工作，加大金融对经济增长的支持力度

加强财政增收节支工作。一是积极培育财源，增强财政供给能力。二是强化税收征管。坚持依法治税，认真组织收入，挖潜堵漏，确保应收尽收、足额入库。加强税收稽查工作，坚决查处和严厉打击各种偷逃漏税行为。三是严格控制财政支出。严格执行《预算法》，强化预算的约束力。加大支出结构调整力度，坚决清理和压缩一般性支出。四是优先保证工资发放和社会保障等重点支出。五是全面推行部门综合预算，切实解决预算内、外资金“两张皮”和财政资金分配“越位”和“缺位”的问题。全面推行政府采购制度，提高财政资金使用效益。六是强化赤字控制。各县（市、区）要制定消化赤字、实现收支平衡的具体计划，不得搞赤字预算，严禁出现新的赤字。七是强化专项资金管理。加强对各类专项资金使用情况的监督检查工作。对已截留、挤占的专项资金，必须制订分步拨付的计划。今年开始，中央、省下达的农业、卫生专项资金，各级财政要实行专户管理，同时,不准截留、挤占新的专项资金，否则从市财政下拨的调度资金中扣还。八是进一步强化“收支两条线”管理。加大审计监督力度，坚决处理私设“小金库”等问题。九是加大政府债务清理力度。树立“还债也是政绩”意识，合理安排偿债资金，逐步解决乡村不良债务问题。

切实做好金融工作。坚持金融部门联席会议制度，加强银企、银农、银贸合作。借鉴福安恒实担保公司和溪柄农村信用社经验，组建担保公司，切实解决中小企业、农民贷款难和银行难贷款等问题。继续加强金融监管，防范和化解金融风险。推进农村信用社改革。不断加强保险业工作。

（九）加强就业和社会保障工作，不断改善人民生活

千方百计扩大就业。认真落实促进再就业的扶持政策，继续从10个方面拓宽再就业渠道，尤其是企事业单位要腾出部分岗位，优先安置“4050”下岗失业人员。加大下岗职工解除劳动关系力度，做好再就业困难群体的援助工作，鼓励下岗失业人员通过灵活多样方式实现再就业，努力促进全市65%以上下岗失业人员实现再就业。同时，努力促进大中专毕业生充分就业。

进一步抓好社会保障工作。继续扩大养老、失业、医疗保险覆盖面，落实城市居民最低生活保障制度，做好“三条保障线”的衔接。继续实施企业离退休人员养老金社会化发放。做好社会福利、救灾救济等民政方面工作，大力发展和规范社会中介组织。

继续提高城乡居民生活水平。必须在发展经济基础上，不断增加城乡居民收入，特别是低收入者的收入。支持农民发展多种经营，搞好庭院经济，努力开辟农民增收的新途径，千方百计增加农民收入。关心离退休老同志、“五老”、受灾户、贫困户、军烈属、伤残军人和其他优抚对象的生活，切实帮助他们解决生活中的困难。

（十）推进精神文明建设和民主法制建设，促进社会事业全面进步

深入开展创建活动，努力提高精神文明建设实效。紧贴社会实际，推动以德治市，全面贯彻落实《公民道德建设实施纲要》，积极推进社会公德、职业道德和家庭美德建设，特别是加强青少年的思想道德建设，引导人们追求更高的思想道德目标。紧贴经济建设，把精神文明建设融入到各项具体工作中，为推动第二层面发展提供强大精神动力和智力支持。紧贴基层需要，采取群众喜闻乐见的方式，开展以文明社区、文明城乡、文明行业为主要内容的群众性精神文明建设创建活动。崇尚科学，反对封建迷信，推进殡葬改革等移风易俗工作。

大力实施“科教兴市”战略，促进社会事业发展。推进科技体制创新，培育发展高新技术企业和民营科技企业，力争实施国家、省级重点科研项目20项。加强科普宣传工作。全面推进素质教育，继续巩固提高“两基”水平，切实解决“两基”遗留问题，大力发展高中教育和职业教育，鼓励社会力量办学。加快师资队伍建设，稳定骨干教师队伍，加快宁德学院、闽东职业技术学院、东侨实验学校筹建步伐。进一步落实人才政策，用好现有人才，引进急需人才，培养高中级人才，特别要用感情、事业、待遇留人，切实解决人才外流问题。加强城乡文化设施建设，继续努力实施文化精品工程，抓好区域特色文化，丰富城乡居民文化生活。力争今年开通宁德电视台公共频道，实现宁德人民广播电台覆盖全市的目标。建立健全以县（市、区）为主的农村卫生分级管理体制，改善农村医疗卫生状况，健全农村卫生服务网络，抓紧建立应对公共卫生事件的应急处理机制，加强对重大疾病的预防控制，努力提高城乡医疗卫生水平。积极推动《全民健身计划纲要》，继续建设全民健身活动点，全面加强竞技体育工作。同时，加快社会科学、新闻出版、外事侨务、档案、地方志、气象、防震减灾、文物保护等各项社会事业发展。

认真实施可持续发展战略，促进经济与人口、资源、环境协调发展。继续落实人口与计划生育工作责任制，稳定低生育水平，全面提高人口素质。推进生态市建设，依法保护和合理使用土地、森林、海洋、矿产和水资源，强化海域使用管理，抓好沿海防护林、江河流域生态林、食用菌产业林、生物多样性保护，绿色工程等生态项目的实施，推进一批生态示范区建设。加强环境监测和污染控制，巩固和扩大“一控双达标”成果，提高城乡人居环境质量。切实加强水土流失综合治理，保护生态环境。扎实推进国土资源市场化步伐，做好土地市场秩序治理整顿工作，全面提升国土资源规划、管理、保护和合理利用水平，继续实现耕地占补平衡。

扎实推进民主法制建设，全力维护社会安定稳定。坚持“有法可依、有法必依、执法必严、违法必究”，推进依法治市进程。认真执行市人大及其常委会的决定、决议，定期报告工作，自觉接受监督。积极支持政协履行职能，重视政协委员参政议政，接受政协民主监督，主动加强同各民主党派、工商联、无党派人士和各人民团体的联系。认真办理人大代表建议、批评和意见以及政协委员提案。健全民主制度，丰富民主形式，扩大基层民主。完善村民自治，扎实抓好村委会换届选举工作，建立和完善村委会民主决策、民主管理、民主监督的机制。完善城镇居民自治，在辖区整合的基础上，建立社区居委会，建设管理有序、文明祥和的新型社区。坚持和完善各种形式的企事业民主管理制度，保障职工的合法权益。认真做好妇女儿童、少数民族和关心下一代工作，发展各项老龄事业，加强残疾人工作，切实维护他们的合法权益。继续实施“四五”普法规划，深入开展“百千万”基层普法依法治理活动，增强全社会的法律素质，尤其要加强公职人员法制观念教育，提高依法办事能力。积极开展法律援助。依法严厉打击各类刑事犯罪活动，坚决扫除社会丑恶现象。坚持打防结合、预防为主，强化社会治安综合治理，切实保障人民群众生命财产安全。加强国家安全工作，坚决打击境内外各种敌对势力的破坏活动，防范和惩治邪教组织的非法活动，依法加强对民族宗教事务和民间信仰活动的管理。认真做好来信来访、人民调解工作，妥善解决久拖不决的上访问题。做好“双拥”支前工作，切实加强国防动员、民兵预备役建设、人民防空等工作，支持驻宁部队和武警部队建设，不断增强军政军民团结。

三、切实加强政府自身建设

加强政府自身建设，是贯彻落实党的十六大精神的重要任务，也是全面完成各项工作任务，顺利推进第二层面发展的基本保证。我们要在市委的领导下，更加自觉地按照“三个代表”重要思想要求，切实增强公仆意识和责任意识，以公正与效率为目标，努力提高依法行政水平，建设全心全意为人民服务的政府。一是与时俱进，紧抓第一要务。二是牢记宗旨，坚持执政为民。三是敢于负责，建设诚信政府。四是依法行政，切实转变职能。五是艰苦奋斗，推进廉政建设。

各位代表，回顾过去，我们信心百倍；展望未来，前景无限美好。让我们紧密团结在以胡锦涛同志为总书记的党中央周围，全面贯彻“三个代表”重要思想，按照党的十六大指引的方向，在省委、省政府和市委的领导下，紧紧依靠全市人民，调动一切积极因素，万众一心，奋发图强，开拓进取，为全面完成今年经济和社会发展的各项任务而努力奋斗！
